# A Brief Introduction to Genomics and Single-cell Analysis for Psychiatric Disorders

**DOI:** 10.14789/ejmj.JMJ25-0003-R

**Published:** 2025-06-04

**Authors:** MASAKI NISHIOKA

**Affiliations:** 1 Department of Psychiatry, Faculty of Medicine, Juntendo University, Tokyo, Japan; 1 Department of Psychiatry, Faculty of Medicine, Juntendo University, Tokyo, Japan; 2 Department of Molecular Pathology of Mood Disorders, Faculty of Medicine, Juntendo University, Tokyo, Japan; 2 Department of Molecular Pathology of Mood Disorders, Faculty of Medicine, Juntendo University, Tokyo, Japan

**Keywords:** genomics, single-cell RNA sequencing, post-mortem brain, bipolar disorder, mosaic variant

## Abstract

Psychiatric disorders, including bipolar disorder (BD) and schizophrenia, represent significant global healthcare challenges. Addressing these issues requires the development of innovative diagnostic and therapeutic strategies grounded in a deeper understanding of the underlying pathological mechanisms. Although the pathophysiology of psychiatric disorders is believed to be rooted in the brain, direct access to the living human brain remains a major limitation. Researchers are actively exploring alternative methods to overcome this challenge. This review examines recent advancements in genomic studies and single-cell RNA sequencing of postmortem brain tissue as promising approaches for understanding psychiatric disorders, with a particular emphasis on BD. Additionally, the potential of induced pluripotent stem cells (iPSCs) as a future extension of genomic research is discussed. By integrating clinical genomic data with cell-type-specific expression profiles, it is possible to identify the specific cell types and brain regions implicated in psychiatric disorders. Further cellular analyses, coupled with drug screening using organoids or neuronal models, hold promise for the development of targeted therapeutic strategies. Coordinated efforts across these areas will contribute to a more comprehensive understanding of the pathological mechanisms underlying psychiatric disorders and facilitate the identification of novel therapeutic opportunities.

## Introduction

Psychiatric disorders, such as bipolar disorder (BD) and schizophrenia, are a global medical challenge^1)^. The medications are effective in alleviating symptoms, but there are many patients with treatment resistance and/or serious adverse effects. This global medical issue should be solved with innovative therapeutic and diagnostic methods based on the understanding of pathological mechanisms. However, we have a big dilemma in psychiatric research to understand the pathological mechanisms of psychiatric disorders. Although the pathological mechanisms of psychiatric disorders are supposed to be in the human brain, the human brain is usually inaccessible in living patients.

Researchers are making various efforts to tackle this dilemma. Some focus on genomic information, considering it the foundational blueprint of organisms. Others utilize induced pluripotent stem cells (iPSCs) to explore detailed aspects of cellular pathology. Additional researchers analyze postmortem brain samples to investigate brain pathology directly, while others adopt non-invasive magnetic resonance imaging (MRI) techniques to observe the human brain in vivo. Each method has its advantages and disadvantages. In this review, I describe the recent genomic studies and single-cell RNA sequencing for postmortem brains as promising approaches for psychiatric disorders with a future perspective using iPSCs as an extension of genomic research.

## Genomic studies

Psychiatric disorders are highly heritable^2)^. For example, the twin-based heritability is estimated as 60% for BD^3)^. Genomes contain the most fundamental information about organisms and are the basis for disease model animals and cultured cells. In addition to biological rationales, the recent advancement of genomic technologies, such as high-throughput sequencers, empowers our investigations^4)^. The question is which kind of genomic information is relevant for psychiatric disorders.

**Figure 1:**
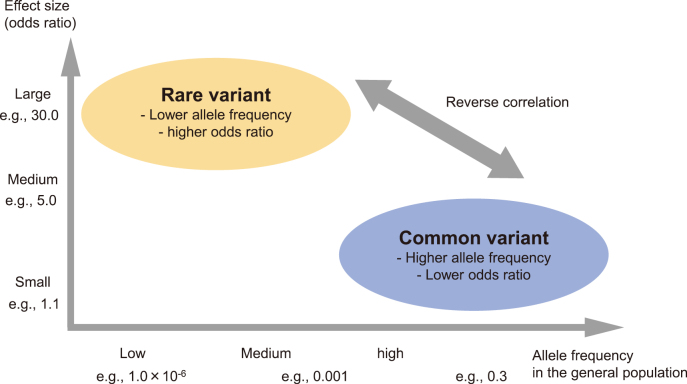
Allele frequency in the general population (x-axis) and effect size (e.g., odds ratio, y-axis)

In psychiatric genomics, Figure 1 is one of the most fundamental frameworks. When plotting the genomic variants with allele frequency in the population on the x-axis and effect size for the disease on the y-axis, there should be an inverse correlation between allele frequency and effect size^5)^. Highly effective variants are negatively selected due to disadvantages to the individual phenotype and tend to be rare, and vice versa. The orange eclipse indicates rare variants, which are rare in the population but have large effect sizes. The blue eclipse indicates common variants commonly observed in the population but have a small effect size. Psychiatric genomics has revealed disease- associated variants following this framework.

## Schizophrenia genomics

Schizophrenia is one of the major psychiatric disorders characterized by hallucinations, delusions, social withdrawal, and cognitive impairment. Recent genomic technology has elucidated rare and common variants associated with schizophrenia. Through rare variants, several genes are associated with schizophrenia, including *GRIA3, XPO7, CUL1, SETD1A, GRIN2A, RB1CC1, SP4, TRIO, CACNA1G, and HERC1*^6)^. These ten genes can be regarded as schizophrenia genes in nearly a monogenic mode. Other copy number variants spanning a genomic region of a mega-base order, including 22q11.21del, 16p11.2dup (proximal), 2p16.3del (*NRXN1*), 15q13.3del, 1q21.1dup/del, 3q29del, 16p11.2del (distal), and 7q11.23dup are associated with schizophrenia^7)^. Here, del indicates deletions, and dup indicates duplications. Besides rare variants, many common variants are associated with schizophrenia. While each common variant alone is not interpretable, the associated common variants are enriched in the genomic regions near synaptic genes, especially post-synaptic genes, indicating the core disease pathogenesis of schizophrenia^8)^.

Notably, both rare and common variants indicate the same biological pathways, namely synaptic genes, especially genes related to voltage-gated cation channel activity. Two genes are surely associated with schizophrenia through common and rare variants: *GRIN2A* and *SP4*^8)^. *GRIN2A* encodes 2A subunit of the NMDA receptor, one of the most investigated targets in molecular neuroscience. SP4 codes a brain-expressed transcription factor. These two genes are at the top of the list for further investigations. Collectively, several hundreds of common variants and several genes with schizophrenia-associated rare variants indicate that schizophrenia is somehow a disease of synaptic disturbances.

## BD genomics

BD, another major mental disorder characterized by cyclical manic and depressive episodes, lags behind schizophrenia in genomic research. Genomic research has found several common variants associated with BD, such as genes related to ion channels, synapse, dopamine, and fatty acid metabolism^9-12)^. The largest GWAS for BD to date^12)^ especially indicates calcium- and dopamine-related pathways for BD. While common variants are intensively investigated, rare variants are relatively under-investigated. Recent genomic studies have revealed a few genes associated with BD through rare variants. Palmer et al. reported *AKAP11* as a bipolar-associated genes^13)^. Kushima et al. reported *ASTN2, DLG2, PCDH15* as promising candidate bipolar-associated genes through short copy number variants^14)^.

In this collective effort, we sought bipolar-associated rare variants by de novo variant analysis^15)^. De novo variants are rare variants observed only in children but not in their parents. De novo means “newly arising” in Latin. Some de novo variants are ultra-rare, observed only in one individual worldwide. We analyzed the largest number of families to date, 354 trios with BD. Compared to control, patients with BD have more rare deleterious de novo variants, especially loss-of-function de novo variants in high pLI genes. “high pLI genes” mean genes that are vulnerable to negative selection due to phenotypic disadvantages by loss-of-function variants. As expected in theory, such high pLI genes are enriched in loss-of-function variants in BD. Among deleterious variants, *KMT2C* and *XKR6* are likely to contribute to bipolar or psychiatric disorders in general. Besides single genes, deleterious de novo variants are enriched in genes related to synapse or calcium ion channel pathways as collective characteristics. These biological pathways are overlapped with those analyzed from common variants for BD^9, 10)^, indicating that the core pathology of BD should reside in these synaptic and ion-channel-related pathways.

Notably, a de novo variant in *KMT2C* was a mosaic variant in BD, while germline variants in *KMT2C* cause a severe developmental disorder (DD), Kleefstra syndrome^16)^. Mosaicness could explain the phenotypic difference between Kleefstra syndrome and BD. Investigating mosaic variants in BD, we have found that deleterious mosaic variants are enriched in DD genes in BD. Among them are recurrent mosaic variants in SRCAP from two independent BD patients. *SRCAP* is one of the DD genes causing Floating-Harbor syndrome^17)^. Both results are unlikely to occur by chance, but the detected mosaic variants are insufficient to argue confidently.

To corroborate this putative result, we subsequently validated it using deep exome sequencing from 231 BD patients to detect mosaic variants with low allele fractions^18)^. Deleterious mosaic variants are again enriched in DD genes in BD. For example, we found a mosaic variant in *ARID2* as the same variant reported as a causative germline variant for Coffin-Siris syndrome, a severe DD^19)^. *ARID2* gene codes a chromatin remodeling protein. The pathogenicity of this variant is established and highly likely to contribute to psychiatric phenotypes. Around four percent of the patients have such mosaic variants, which is a 5.6-fold enrichment from expectation, indicating a possible genomic stratification. This enrichment is not observed in germline de novo variants in BD nor mosaic and germline de novo variants in control trios.

Extending our investigation to mitochondrial mosaic variants, or heteroplasmic variants, we found heteroplasmic variants in tRNA regions are enriched in BD. The proportion of heteroplasmic tRNA variants in BD trios is higher than in the theoretical estimate and control trios. The enrichment is around 2.8-fold. The enrichment becomes stronger when limited to deleterious tRNA variants, showing 4.7-fold enrichment. These results indicate that heteroplasmic tRNA variants, especially deleterious tRNA variants, are associated with BD. Around three percent of BD patients have deleterious heteroplasmic tRNA variants, indicating possible genomic stratification. Notably, recurrent m.3243A > G variants, the major causative variants for mitochondrial disease MELAS^20)^ as deleterious tRNA variants, are observed in two independent BD patients. The allele fractions were 5 and 12 percent, lower than MELAS's 20~%. Besides peripheral tissue investigations, m.3243A > G variants were recurrently observed in the postmortem brain tissues of two of 15 BD patients but not observed in the brains of 14 controls^21)^. This consistency supports the association between m. 3243A > G variants and BD.

**Figure 2:**
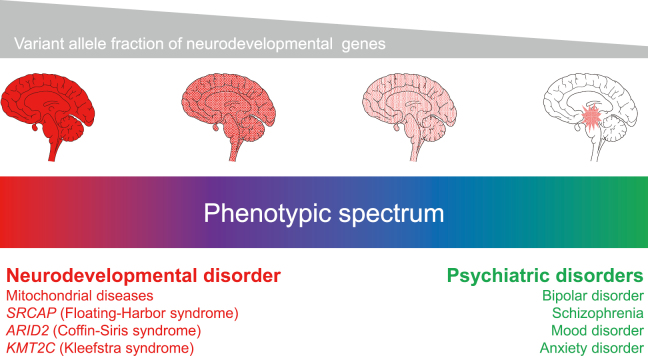
Phenotypic spectrum by allele fractions of neurodevelopmental genes. Individual phenotypes are affected by the allele fractions of neurodevelopmental genes. Higher variant allele fractions or germline variants cause neurodevelopmental disorders such as mitochondrial diseases, while lower variant allele fractions contribute to psychiatric disorders such as bipolar disorder.

**Figure 3:**
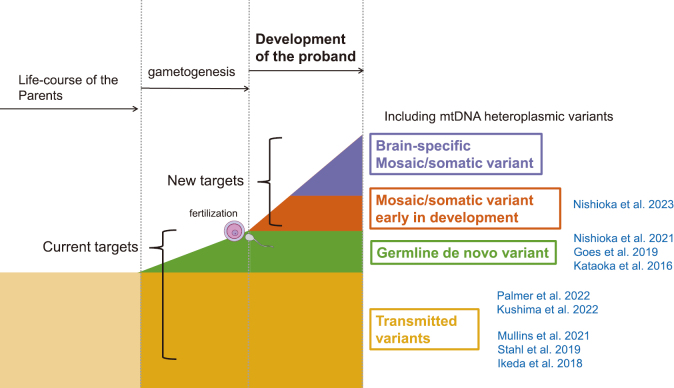
Multi-stage variant accumulation model. Yellow indicates transmitted variants from the parents. Green indicates germline de novo variants arising during gametogenesis in the parents. Orange and purple indicate mosaic variants, new targets of psychiatric genomics. These four classes of variants accumulate in the probands’ brain, contributing to psychiatric disorders.

**Figure 4:**
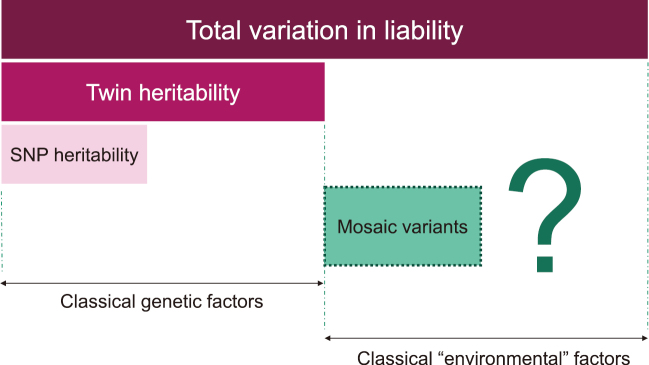
Liability model including mosaic variants. Mosaic variants are not included in classical genetic factors as estimated from twin-based heritability calculations. Mosaic variants could explain a part of factors classically attributed to “environmental” factors.

These results lead to a hypothesis of possible phenotypic spectrum by mosaic allele fractions of neurodevelopmental genes (Figure 2). While deleterious germline or high allele fraction variants in neurodevelopmental genes result in severe DDs, mosaic variants in the same position with moderate allele fractions lead to psychiatric disorders such as BD. This model can be extended to mitochondrial variants and mitochondrial diseases. Combining classical genetic factors, I propose a multi-stage variant accumulation model for psychiatric disorders^22)^ (Figure 3). Previous genomic investigations have elucidated the germline variants associated with BD^9, 11, 13)-15, 23, 24)^. The contribution of mosaic variants to BD is worth investigating to explain the remaining part of the liability of BD^22)^. Note that the possible contribution of mosaic variants is not categorized as traditional genetic factors but could explain a part traditionally supposed to be “environmental” in a classical genetic vs environmental framework because mosaic variants are not included in the heritability estimates (Figure 4).

## Single-cell analysis

Single-cell analysis, here single-cell RNA sequencing, is one of the most powerful methods in modern biology. This method can analyze comprehensive landscapes of gene expression in thousands of single cells. The human cell atlas project, the next big bio-project after the human genome project, comprehensively catalogs human cells’ gene expression with this single-cell technology. In reality, human tissues can be analyzed at the cell-nucleus level because cells cannot be extracted from frozen tissues. Thus, single-nucleus RNA sequencing (snRNA-seq) is a feasible approach^25, 26)^. While the human brain is one of the most difficult targets among human tissues due to its low availability and high complexity, snRNA-seq can provide new basic knowledge for the human brain cell types^25, 27-31)^, including inter-species differences between humans and other animals^32-34)^. The human brain is as mysterious as the universe. The snRNA-seq is a new telescope for the unknown.

Publications of snRNA-seq for psychiatric disorders are relatively limited due to the difficulty of postmortem brain sample collection and experimental costs^35-41)^ (Table 1). Indeed, no reports of snRNA-seq case-control comparisons for BD have been published as of 2024. Besides the low availability of postmortem brain samples, psychiatric disorders cast another problem of which brain region to investigate, and we have no definitive answer to this question. While case-control comparisons using various brain regions are desirable, snRNA-seq for various disorders for various brain regions is unrealistic, given the low availability of samples and experimental costs. Thus, a complementary approach of integrating genomic data and single-cell RNA-seq data from control samples to infer responsible brain regions and cell types is needed.

**Table 1:** Major snRNA-seq studies for psychiatric disorders

Disease	Authors	Titles	Journal	Year	Sample size	Brain region
Schizophrenia	Batiuk et al.	Upper cortical layer–driven network impairment in schizophrenia	Science Advances	2022	9 vs 14	dorsolateral prefrontal cortex (BA9)
	Puvogel et al.	Single-nucleus RNA sequencing of midbrain blood-brain barrier cells in schizophrenia reveals subtle transcriptional changes with overall preservation of cellular proportions and phenotypes	Molecular Psychiatry	2022	15 vs 14	blood-brain barrier of midbrain
	Ruzicka et al.	Single-cell multi-cohort dissection of the schizophrenia transcriptome	Science	2024	65 vs 75	prefrontal cortex
	Ling et al.	A concerted neuron–astrocyte program declines in ageing and schizophrenia	Nature	2024	94 vs 97	dorsolateral prefrontal cortex (BA46)
Autism spectrum disorder	Velmeshev et al.	Single-cell genomics identifies cell type–specific molecular changes in autism	Science	2019	15 vs 16	prefrontal cortex (PFC) and anterior cingulate cortex (ACC)
	Wamsley et al.	Molecular cascades and cell type–specific signatures in ASD revealed by single-cell genomics	Science	2024	33 vs 30	frontal cortex
Major depression	Nagy et al.	Single-nucleus transcriptomics of the prefrontal cortex in major depressive disorder implicates oligodendrocyte precursor cells and excitatory neurons	Nature Neuroscience	2020	17 vs 17	dorsolateral prefrontal cortex (BA9)
	Maitra et al.	Cell type specific transcriptomic differences in depression show similar patterns between males and females but implicate distinct cell types and genes	Nature Communications	2023	37 vs 34	dorsolateral prefrontal cortex (BA9)

## Integrating snRNA-seq and genomics data

**Figure 5:**
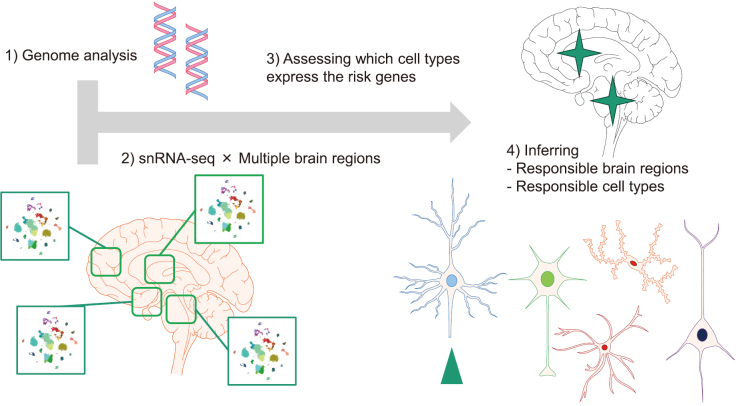
Integrating genome and snRNA-seq analysis to explore responsible brain cel types. Single-cell data of control samples provides characteristically expressed genes in each cell type, including non-famous or unknown cell types. Assessing which cell type expresses the risk genes more than other cell types can infer the candidate responsible cell type for specific disorders. We can infer the responsible brain regions and cell types by extending this analysis to various brain regions.

Single-cell data of control samples provide characteristically expressed genes in each cell type, including unknown cell types. Assessing which cell type expresses the risk genes more than other cell types can infer the candidate responsible cell type for specific disorders. We can infer the responsible brain regions and cell types by extending this analysis to various brain regions (Figure 5).

As an example, we assessed which tissue and cell type preferentially express the putative risk genes with single-cell RNA-seq data to infer the responsible cell types in BD^15)^. The genes hit by deleterious de novo variants in our study are more expressed in the anterior cingulate cortex (ACC) than in other brain tissues and organs. These genes are more expressed in excitatory neurons than in other brain cell types in the ACC. Excitatory neurons are more susceptible to BD genetic factors than other brain cell types. One subtype of excitatory neuron expresses the largest number of the genes hit by deleterious de novo variants. This neuron characteristically expresses DD genes and GWAS index genes. This is intriguing because we found somatic variants in BD are enriched in DD genes, as explained before. This subtype of excitatory neuron expresses *CACNA1C*. This analysis supported the relevance of *CACNA1C*, which encodes a subunit of a voltage-gated calcium channel, Cav1.2, and is one of the most investigated genes for BD as the first reported BD-associated genes^42)^. O’Connell et al. analyzed the enrichment of BD risk gene expression using the largest BD GWAS^12)^ and the largest human brain single-nucleus RNA-seq dataset to date^28)^. The BD risk genes expressed preferentially in hippocampal pyramidal neurons and interneurons of the prefrontal cortex/hippocampus in their analysis.

This formula can be applied to various disorders other than BD. When applying this formula to schizophrenia risk genes and various brain region single-cell data from mice, the schizophrenia risk genes are enriched in the genes expressed in excitatory neurons, especially those in the telencephalon^7)^. Given the limited number of human brain single-cell data, mouse single-cell data is a feasible surrogate at present. This cell-type enrichment is shared between common and rare variants, indicating overlapped biological mechanisms between common and rare variants.

The best practice of this approach is Parkinson’s disease and substantia nigra^43)^. Expectedly, the Parkinson’s disease risk genes are enriched in the genes expressed in dopaminergic neurons in the substantia nigra. This data is a positive control result of our formula since the dopaminergic neuron is known to be particularly affected in PD. One subtype of dopaminergic neurons expressing SOX6 and AGTR1 is especially decreased in PD. The upregulation of target genes of TP53 and NR2F2 in this neuron in PD indicates therapeutic target pathways. This single-cell data provides further information about cellular pathology beyond existing knowledge.

## iPSC-based approach

I have illustrated how genomic information can lead to pathological insight. But medicine needs therapeutics. One promising approach is using iPSC-induced nervous system cells, simulating the pathological mechanisms to test potential drugs. Parkinson’s disease studies benefit from iPSC- derived dopaminergic neurons. A question in psychiatry is which brain region or cell type is the best material for specific disorders, such as schizophrenia and BD.

For example, BD is characterized by a thalamic volume reduction associated with lithium treatment^44)^. The thalamus is a promising brain region for BD. One group at Yale University reported organoids simulating the human thalamus^45, 46)^. These thalamic organoids are candidate models simulating the thalamic pathology in BD. By generating thalamic organoids from the peripheral sample of BD patients with relevant genomic variants, we can develop an in vitro BD model. This can be used to simulate BD cellular pathology and assess potential medications for BD. Besides thalamic organoids for BD, iPSC-derived organoids/neurons simulating other brain regions or cell types are expected for other psychiatric disorders.

As to relevant variants for BD, mitochondrial heteroplasmic variants, including m.3243A > G variants, are expecting candidates, as explained before. Targeting mitochondria can extend the therapeutic development from rare mitochondrial diseases to common mood disorders, including BD. Indeed, several compounds are tested for mitochondrial diseases, some of which could be repurposed to BD. For example, Kubota-Sakahshita et al. explore drugs to fix or modulate mitochondrial functions^47)^. I expect we will have mitochondria-targeted therapy for mood disorders and other medical conditions in the future.

Further studies are needed for this approach. Current risk gene information is far from perfect, and novel variants need to be detected. Phenotypic information, such as treatment resistance, will be helpful for patient stratification. Single-cell data using the human brain is accumulating, but various brain regions remain to be explored. In particular, subcortical regions need further investigation. For an iPSC-based approach, we need various organoids or induced cell types simulating specific brain regions or cells. The main issue is which cell type is the best for specific diseases. While many issues remain to be solved, the coordination of these efforts will elucidate the pathogenesis of psychiatric disorders and new therapeutic development.

## Conclusion

I have briefly introduced the recent advancement of genomic and single-cell analysis for psychiatric disorders, focusing on BD. Integrating clinical genomic analysis and cell-type-specific expression data can infer responsible cell types and brain regions for psychiatric disorders. The inferred cellular pathology can be simulated by iPSC- induced organoids or neurons derived from patients with relevant variants. Further cellular analysis and drug examination using the generated organoids/neurons can lead to therapeutic development. Future efforts coordinating these efforts will elucidate the pathological mechanisms of psychiatric disorders and new therapeutic opportunities.

## Author contributions

MN wrote and checked the manuscript

## Conflicts of interest statement

MN belongs to the Department of Molecular Pathology of Mood Disorders, Faculty of Medicine, Juntendo University, a joint laboratory of Juntendo University and Sumitomo Dainippon Pharma.
